# Image analysis in posttreatment non-small cell lung cancer surveillance: specialists’ interpretations reviewed by the thoracic multidisciplinary tumor board

**DOI:** 10.1186/s40248-019-0198-z

**Published:** 2019-12-04

**Authors:** Franco Gambazzi, Lukas D. Frey, Matthias Bruehlmeier, Wolf-Dieter Janthur, Juerg Heuberger, Andres Spirig, Richard Williams, Roland Zweifel, Bettina Boerner, Gabrielo M. Tini, Sarosh Irani

**Affiliations:** 1 Clinic of Thoracic Surgery, Cantonal Hospital Aarau, Tellstrasse, CH-5001 Aarau, Switzerland; 2 Institute of Nuclear Medicine and PET-Center, Cantonal Hospital Aarau, Tellstrasse, CH-5001 Aarau, Switzerland; 3 Clinic of Oncology, Cantonal Hospital Aarau, Tellstrasse, CH-5001 Aarau, Switzerland; 4 Clinic of Radio-Oncology, Cantonal Hospital Aarau, Tellstrasse, CH-5001 Aarau, Switzerland; 5 Department of Radiology, Cantonal Hospital Aarau, Tellstrasse, CH-5001 Aarau, Switzerland; 6 Institute of Pathology, Cantonal Hospital Aarau, Tellstrasse, CH-5001 Aarau, Switzerland; 7 Clinic of Pulmonary and Sleep Medicine, Cantonal Hospital Aarau, Tellstrasse, CH-5001 Aarau, Switzerland

**Keywords:** Posttreatment lung cancer surveillance, Agreement on imaging, Multidisciplinary tumor board

## Abstract

**Background:**

Data show that the initial specialist’s image interpretation and final multidisciplinary tumor board (MTB) assessment can vary substantially in the pretherapeutic cancer setting. The aim of this *post hoc* analysis was to investigate the concordance of the specialist’s and MTB’s image interpretations in patients undergoing systematic posttreatment lung cancer image surveillance.

**Methods:**

In the initial prospective study, lung cancer patients who had received curative-intent treatment were randomly assigned to undergo either contrast-enhanced computed tomography (CE-CT) or integrated 18^F^-fluorodeoxyglucose positron emission tomography-computed tomography (PET-CT). Imaging was performed every 6 months for 2 years, and all imaging studies were finally assessed by our MTB. This *post hoc* analysis assessed differences between the initial specialist’s image interpretation and the final MTB’s image interpretation.

**Results:**

In 89 patients, 266 imaging studies (129 PET-CT, 137 CE-CT) were analyzed. In 87.2% (88.4, 86.1%) of the studies, complete concordance was found. Out of the 12.8% (11.6, 13.9%) with discordant results, 7.5% (6.9, 8.0%) had implications for alterations in patient management (major disagreements).

Twenty major disagreements were detected in 17 study patients. Retrospectively, in eight out of these 17 (47%) patients, in contrast to the MTB’s view, the specialist’s interpretation was more appropriate, whereas in nine out of 17 patients (53%), the MTB’s interpretation was more accurate.

**Conclusions:**

In an experienced MTB, the agreement between imaging specialists and the rest of the MTB with regard to the interpretation of images is high in a setting of posttreatment lung cancer image surveillance. It seems that in cases of disagreements, the rates of more accurate interpretation are well balanced between imaging specialists and the MTB.

**Trial registration:**

ISRCTN16281786, Date 23. February 2017.

## Background

There is increasing evidence that the pretherapeutic presentation of cancer patients to multidisciplinary tumor boards (MTBs) frequently impacts patient management not only with regard to the adherence to guidelines [1, 2] but also with regard to changing cancer staging [3, 4] and therapeutic procedures [1, 5–9]. Particularly for patients with non-small cell lung cancer (NSCLC), evaluation by a MTB even seems to improve patient survival [10–12]. With regard to image interpretation, disagreements have been identified between the original radiologist report and the MTB consensus on the results of the image analysis in up to 30% of cases [13].

Systematic surveillance after curative-intent treatment of NSCLC is widely recommended, though the modality and length of cancer surveillance is neither well studied nor uniformly agreed on in guidelines [14]. Posttherapeutic image analysis in NSCLC patients is challenging in several ways. First, in most cases after therapeutic interventions, there are residual findings such as scars or effusions. Second, in contrast to the pretherapeutic state, the majority of images do not show any signs of cancer (low pretest probability). Finally, in the case of cancer recurrence, the particular image findings can be discrete and difficult to see.

In contrast to pretherapeutic image analysis, data on the rate of concurrence between specialists’ and MTBs’ interpretations of posttherapeutic images from NSCLC patients are lacking.

The aim of the current study was to compare specialists’ and an MTB’s interpretations of contrast-enhanced computed tomography (CE-CT) and integrated F-fluorodeoxyglucose positron emission tomography-computed tomography (PET-CT) images in the context of posttherapeutic cancer surveillance of NSCLC patients.

## Methods

This study was a *post hoc* analysis of a randomized pilot study that has recently been published [15]. The study protocol of this prospective study was approved by our Ethics Committee (Kantonale Ethikkommission Aargau, Switzerland, Protocol No 2011/045). Written informed consent was obtained from all participants. This clinical trial was registered (ISRCTN16281786).

In brief, NSCLC patients after curative-intent treatment were 1:1 randomized to structured CE-CT or PET-CT surveillance between October 11, 2011, and August 29, 2014. The surveillance examinations were performed at 6-month intervals during the two-year follow up period. CE-CT and PET-CT images were interpreted by senior specialists. The particular specialists were consistently members of the MTB. All surveillance examinations were discussed at our weekly MTB meeting. By definition, the study surveillance was finished as soon as the MTB deemed an image finding suspicious, and further diagnostic or therapeutic steps were considered necessary.

Our institution serves Canton Aargau, which includes approximately 600,000 people. Our weekly thoracic MTB meetings started on August 6, 2010, and included members from thoracic surgery, pulmonology, medical oncology, nuclear medicine, radiology, pathology and radiation oncology departments. The participating health care professionals have been members of the MTB for many years. After the weekly meeting, an online summary of findings and recommendations is sent to all members of the MTB for approval. Fifteen to 25 cases are discussed at our thoracic MTB meeting each week. The MTB adheres to the “all cases” concept [16]. This means that without exception all patients of our institution suffering from non-small cell lung cancer are discussed at our MTB to define the initial therapy, the changes in therapy or to discuss surveillance studies.

In the current study, reports from surveillance examinations from radiologists and nuclear medicine specialists were compared with the results of the MTB protocol. All surveillance imaging studies are reviewed by the MTB. The particular definitions of agreement and disagreement, respectively, were predefined (see Table 1). Patients who showed symptomatic recurrence before the first surveillance study was scheduled were not considered.

**Table 1:** Definitions

	Definition
Agreement	Complete agreement between specialist and MTB
positive	a radiological finding is interpreted as suspicious by both
negative	agreement that image is without suspicious findings
Disagreement	Disagreement between specialist and MTB
major	disagreement implies management alteration
diagnostic^a^	alteration in diagnostic procedures due to MTB's interpretation
therapeutic^a^	alteration in therapeutic procedures due to MTB's interpratation
benign to malignant^a^	increase in level of suspicion due to MTB’s interpretation
malignant to benign^a^	decrease in level of suspicion due to MTB’s interpretation
minor	disagreement implies no management alteration

*MTB* multidisciplinary tumor board

^a^one or more options per case possible

In a second step, it was retrospectively considered whether the interpretation of the specialist or the MTB was ultimately more appropriate.

Statistica 10.0 software (StatSoft, Inc., Tulsa, OK) was used for the statistical analyses. Absolute numbers (percentages) and medians (interquartile ranges, IQRs) were used to describe the study population and the rates of agreement and disagreement, respectively. Due to the descriptive nature of the current study and the lack of a power analysis, a comparison of study results between imaging procedures was not pursued. To identify differences between groups of patients, Mann-Whitney U-test for independent samples or the Chi-square test was used where appropriate. A *p* less than 0.05 was considered statistically significant.

## Results

Due to symptomatic recurrence before the first surveillance study, out of 96 patients included in the original prospective study, seven did not have any surveillance studies. Therefore, the images of a total of 89 patients, including 130 PET-CTs of 45 patients and 138 CE-CTs of 44 patients, respectively, were analyzed. One PET-CT and one CE-CT were excluded from further analysis because the written final imaging report was dated after the corresponding MTB meeting took place so a final number of 266 scans were analyzed. The baseline characteristics of the study population are summarized in Table 2.

**Table 2:** Baseline characteristics

	Total number of patients *n* = 89	PET-CT *n* = 45	CE-CT *n* = 44
Age, years	65.3 (57.6-73.2)	67.6 (59.8-74.5)^*^	60.8 (56.6-70.1)^*^
Gender, female	27 (30.3)	16 (35.6)	11 (25)
Adenocarcinoma	57 (64)	30 (66.7)	27 (61.4)
NSCLC stage			
I	44 (49.4)	21 (46.7)	23 (52.3)
II	24 (26.9)	14 (31.1)	10 (22.7)
III	18 (20.2)	8 (17.8)	10 (22.7)
IV^a^	3 (3.4)	2 (4.4)	1 (2.3)
Therapy			
Surgery alone	58 (65.2)	31 (68.9)	27 (61.4)
Surgery + adjuvant chemoth.	19 (21.4)	7 (15.6)	12 (27.3)
Surgery + neoadjuvant chemoth.	7 (7.8)	4 (8.9)	3 (6.8)
Radiotherapy +/- chemoth.	2 (2.3)	1 (2.2)	1 (2.3)
Other	3 (3.4)	2 (4.4)	1 (2.3)
Total number of surveillance studies	266	129	137

Data presented as medians (IQRs) or numbers (%)

*PET-CT* integrated F-fluorodeoxyglucose positron emission tomography-computed tomography, *CE-CT* contrast-enhanced computed tomography, *NSCLC* non-small cell lung cancer

^*^p = 0.037 PET-CT vs. CE-CT

^a^solitary brain metastasis

The results of the analyses by the specialists and the MTB and the agreement between the two regarding the 266 images are summarized in Table 3.

**Table 3:** Agreement between specialist’s and MTB’s image interpretations

Outcome^a^	Total number of surveillance studies *n* = 266	PET-CT *n* = 129	CE-CT *n* = 137
Agreement	232 (87.2)	114 (88.4)	118 (86.1)
positive	39 (14.7)	22 (17.1)	17 (12.4)
negative	193 (72.6)	92 (71.3)	101 (73.7)
Disagreement	34 (12.8)	15 (11.6)	19 (13.9)
major	20 (7.5)	9 (6.9)	11 (8.0)
diagnostic	17 (6.4)	9 (6.9)	8 (5.8)
therapeutic	4 (1.5)	3 (2.3)	1 (0.7)
benign to malignant	7 (2.6)	3 (2.3)	4 (2.9)
malignant to benign	10 (3.7)	5 (3.8)	5 (3.6)
minor	14 (5.3)	6 (4.6)	8 (5.8)

*MTB* multidisciplinary tumor board, *PET-CT* integrated F-fluorodeoxyglucose positron emission tomography-computed tomography, *CE-CT* contrast-enhanced computed tomography

^a^Definitions: see Table 1, data presented as number (%). No statistically significant differences between PET-CT and CE-CT were observed

Additional details about the disagreements are summarized in Table 4.

**Table 4:** Summary of disagreements

	Number of events	MTB opinion in contrast to specialist’s recommendation
Major		
diagnostic^a^	4	other control interval
	9	no alteration of surveillance plan
	4	stop surveillance, further diagnostic or therapeutic steps
therapeutic^a^	1	no therapy of presumed incomplete resection
	1	resection of suspicious lymph node
	1	resection of suspicious pleural thickening
	1	resection of pulmonary nodule
benign to malignant^a^	3	interpretation of pulmonary lesion as suspicious
	1	interpretation of pleural lesion as suspicious
	3	interpretation of lymph node as suspicious
malignant to benign^a^	1	interpretation of liver lesion as less suspicious
	2	interpretation of bone lesion as less suspicious
	5	interpretation of pulmonary lesion as less suspicious
	2	interpretation of lymph node as less suspicous
Minor	4	lymph node size
	2	pericardial effusion
	1	postoperative lesion
	4	lung lesions
	2	bone lesions
	1	liver lesion

*MTB* multidisciplinary tumor board

^a^one or more options per case possible, e.g. resection of suspicious mediastinal lymph nodes

Twenty major disagreements were detected in 17 different patients. Retrospectively, in eight out of these 17 (47%) patients, in contrast to MTB’s view, the specialist’s interpretation turned out to be more appropriate. In none of these cases there was a potentially curable cancer manifestation missed. On the other hand, in nine out of 17 patients (53%), the analysis MTB was retrospectively determined to have been more accurate (data not shown). With regard to age, sex, the number of surveillance studies, cancer stage and neoadjuvant pretreatment, we did not find significant differences between these 17 patients and the 72 patients of the entire group (*p* = 0.754, 0.279, 0.261, 0.201 and 0.735, respectively).

## Discussion

In the current study we found complete concordance between the initial specialist’s image interpretation and the final MTB’s image interpretation in 87.2% of the studies. Out of the discordant studies, 7.5% had implications for alterations in patient management. Retrospectively, in cases of disagreements, the rates of more accurate interpretation were well balanced between imaging specialists and the MTB.

Discussion by the MTB can change the therapeutic management plan of cancer patients in up to 52% of cases [8]. In approximately 10 [6, 7, 17] to 45% [8] of cases, changes are made due to review of the images by the MTB. In the case of lung cancer, several studies observed a cancer survival benefit when treatment plans came from the MTB rather than from individual physicians [18, 19]. However, few data exist regarding the impact of image interpretation revision by the MTB on patient outcomes. Recently, Schmidt et al. [9] have shown in a cohort of patients with lung and esophageal cancer that the MTB recommends changing therapeutic plans in a substantial proportion of patients (24%) due to a change in staging. In most cases, this was achieved by reviewing diagnostic images.

Data regarding image interpretation agreement in the surveillance setting are limited. Li et al. [20] found excellent agreement between two radiologists who evaluated CT scans after stereotactic body radiotherapy (Kappa values 0.68 to 1). In contrast, Gierada et al. [21] found moderate interobserver agreement regarding the interpretation of low-dose lung cancer screening CT scans (Kappa 0.58 to 0.64).

Posttreatment imaging surveillance after lung cancer therapy is costly in terms of resources [22], and efforts should be made to improve the evidence provided by this procedure. This includes determining the interrater agreement regarding the interpretation of the images. In our lung cancer treatment program, we therefore systematically review all images at our MTB meetings in both the pre- and posttreatment settings. The overall disagreement rate of 12.8% in our current study is less than the 30% reported by Masch et al. [13]. In that study, pretherapeutic radiological reports were reviewed by the hepatobiliary tumor board. Nevertheless, in only 8% of their caseschanges in the subsequent patient management occurred due to the findings of the MTB, which is quite similar to the 7.5% observed in our study. In a study of a pediatric MTB, [3] changes in the management of the patients were made in 7.6% of cases based on a review of pretherapeutic radiology images. Lee et al. [17] reported in a study of a gynecologic MTB that the review of images resulted in a change in interpretation in 10.6% of cases, 3.5% of these changes resulted in a change in the treatment plan. The 7.5% we observed in our study is higher probably because we considered all types of management changes rather than only treatment plan changes.

We were not able to identify differences between patients involved and not involved in disagreements between the specialists and the MTB. This might be the consequence of the relatively small number of patients. The fact that in most cases in the posttherapeutic setting no cancer is visible and treatment residues are comparable between patients potentially contributes to this observation.

In our population, the further follow up of the 17 patients regarding whose imaging studies major disagreements occurred revealed the interesting finding that the accuracy of the specialist’s and MTB’s interpretations were well balanced. In approximately 50% of cases in which major disagreements occurred, the initial interpretation of the specialist was retrospectively determined to be more accurate than interpretation of the MTB and vice versa. It is important to stress that no curable cancer was missed in any retrospective view. Although the observed disagreements led to changes in management, no severe management errors occurred, particularly in those cases in which the interpretation of the MTB was retrospectively determined to be less accurate. This indicates that, most likely in cases of ambiguous images, the safer procedure is preferred by the MTB so as not to miss a potentially curable cancer recurrence.

The limitations of our current study include the relatively small number of cases. In particular the low numbers of factors such as radiotherapy that potentially interfere in a relevant way with image interpretation make a more detailed analysis impossible. The strengths include the initial prospective inclusion of patients and the homogenous management of all patients in our structured surveillance program, which is part of the MTB. Furthermore, we believe that the competence of the MTB of our institution is high particularly due to our “all case” concept. This concept ensures both an ideal initial therapy conception and adequate therapy alterations in patients suffering from non-small cell lung cancer.

## Conclusions

In conclusion, it seems that the rate of disagreements in the interpretation of images in the context of structured posttherapeutic lung cancer surveillance is low. Disagreements occur in roughly 10 % of examinations, a rate that is comparable to those published in the pretherapeutic cancer context. In addition, we believe that both imaging specialists and the MTB can learn from each other in the context of surveillance. For this reason, we strongly believe that interpretation of lung cancer imaging surveillance should be part of the role of the MTB.

## Data Availability

The datasets analysed during the current study are available from the corresponding author on request.

## Abbreviations

CE-CT: Contrast-enhanced computed tomography
IQR: Interquartile ranges
MTB: Multidisciplinary tumor board
NSCLC: Non-small cell lung cancer
PET-CT: Integrated 18F-fluorodeoxyglucose positron emission tomography-computed tomography
